# Supplementary material for: Decreasing prevalence of contamination with extended-spectrum beta-lactamase-producing Enterobacteriaceae (ESBL-E) in retail chicken meat in the Netherlands
Source: PLoS One. 2019 Dec 31;14(12):e0226828. doi: 10.1371/journal.pone.0226828 (PMC6938319; doi:10.1371/journal.pone.0226828)
Supplement: S1 Table — (DOCX) [file pone.0226828.s001.docx]

**S1 Table. Number of samples taken per month, per supermarket chain and per method of farming.**

|  |  | SC1 | | SC2 | | SC3 | | SC4 | |  |
| --- | --- | --- | --- | --- | --- | --- | --- | --- | --- | --- |
| Period | Month | Conventional | Free range | Conventional | Free range | Conventional | Free range | Conventional | Free range | Total per sampled month |
| Period 14 | 1 |  |  | 3 |  | 5 |  | 2 |  | 10 |
|  | 2 | 8 | 3 | 1 | 5 | 8 |  | 1 | 2 | 28 |
|  | 3 |  |  |  |  | 1 |  |  |  | 1 |
|  | 4 |  |  |  |  | 3 |  |  |  | 3 |
|  | 5 |  |  | 2 | 1 | 3 |  | 1 | 1 | 8 |
|  | 6 | 2 |  | 2 |  | 6 |  | 3 |  | 13 |
|  | 7 | 2 | 1 | 3 | 3 |  |  | 2 |  | 11 |
|  | 8 | 3 |  | 4 | 1 | 12 | 1 | 4 | 2 | 27 |
|  | 9 | 7 | 2 | 5 | 1 | 6 |  | 6 | 4 | 31 |
|  | 10 | 2 | 1 |  |  | 3 | 1 | 2 | 1 | 10 |
| Period 15 | 18 | 1 |  | 7 | 2 | 6 | 2 | 2 | 1 | 21 |
|  | 19 | 19 | 8 | 28 | 15 | 27 | 13 | 22 | 16 | 148 |
|  | 20 | 16 | 7 |  |  | 1 | 2 | 6 | 3 | 35 |
|  | Total period 14 | 24 | 7 | 20 | 11 | 47 | 2 | 21 | 10 | 142 |
|  | Total period 15 | 36 | 15 | 35 | 17 | 34 | 17 | 30 | 20 | 204 |
|  | Total period 14 + 15 | 60 | 22 | 55 | 28 | 81 | 19 | 51 | 30 | 346 |
|  | Total per SC | 82 | | 83 | | 100 | | 81 | |  |
| Abbreviation: SC, supermarket Chain; | | | | | | | | | | |
