# Supplementary material for: Decreasing prevalence of contamination with extended-spectrum beta-lactamase-producing Enterobacteriaceae (ESBL-E) in retail chicken meat in the Netherlands
Source: PLoS One. 2019 Dec 31;14(12):e0226828. doi: 10.1371/journal.pone.0226828 (PMC6938319; doi:10.1371/journal.pone.0226828)
Supplement: S3 Table — (DOCX) [file pone.0226828.s003.docx]

| **Plasmid** | **Frequency**  **n=974** | **Prevalence in ESBL-E isolates (%)** |
| --- | --- | --- |
| IncFIB | 173 | 80.1 |
| Col | 167 | 77.3 |
| IncI1 | 149 | 69.0 |
| IncFII | 120 | 55.6 |
| p0111 | 68 | 31.5 |
| IncFIC(FII) | 63 | 29.2 |
| IncB/O/K/Z | 45 | 20.8 |
| IncX1 | 38 | 17.6 |
| IncFIA | 26 | 12.0 |
| IncX3 | 21 | 9.7 |
| IncI2 | 19 | 8.8 |
| IncQ1 | 13 | 6.0 |
| IncX4 | 13 | 6.0 |
| IncY | 10 | 4.6 |
| IncA/C2 | 8 | 3.7 |
| IncHI1B | 8 | 3.7 |
| IncHI2 | 7 | 3.2 |
| IncHI2A | 7 | 3.2 |
| TrfA | 7 | 3.2 |
| IncR | 5 | 2.3 |
| IncHI1A | 4 | 1.9 |
| IncN | 3 | 1.4 |

**S3 Table. Detected plasmid replicon families and the number of ESBL-E isolates from retail chicken meat they were detected in.**
