# Supplementary material for: Decreasing prevalence of contamination with extended-spectrum beta-lactamase-producing Enterobacteriaceae (ESBL-E) in retail chicken meat in the Netherlands
Source: PLoS One. 2019 Dec 31;14(12):e0226828. doi: 10.1371/journal.pone.0226828 (PMC6938319; doi:10.1371/journal.pone.0226828)
Supplement: S4 Table — (DOCX) [file pone.0226828.s004.docx]

**S4 Table. Frequency of clonality within the three most common multilocus sequence types with the median, minimum and maximum time between isolates for related and unrelated isolates within the ST.**

|  |  |  |  | Clonally related comparisons | | | | Non-clonally related comparisons | | |  |
| --- | --- | --- | --- | --- | --- | --- | --- | --- | --- | --- | --- |
| ST | No. Comparisons | Clonally related | % Clonally related | Med t | Min t | Max t | Med t | | Min t | Max t | |
| ST 117 | 528 | 24 | 4.5 | 6.5 | 0 | 346 | 276 | | 0 | 535 | |
| ST 10 | 153 | 8 | 5.2 | 8 | 0 | 21 | 286 | | 1 | 558 | |
| ST 602 | 105 | 87 | 82.9 | 94 | 0 | 226 | 374 | | 0 | 530 | |
| Abbreviations: ST, sequence type; No, number of; t, time between isolates; med, median; min, minimum; max, maximum | | | | | | | | | | |  |
