# Supplementary material for: Decreasing prevalence of contamination with extended-spectrum beta-lactamase-producing Enterobacteriaceae (ESBL-E) in retail chicken meat in the Netherlands
Source: PLoS One. 2019 Dec 31;14(12):e0226828. doi: 10.1371/journal.pone.0226828 (PMC6938319; doi:10.1371/journal.pone.0226828)
Supplement: S5 Table — Within the pairwise comparisons all ST were the same except for an isolate with ST43 which was clonally related to an isolate of ST8567. (DOCX) [file pone.0226828.s005.docx]

**S5 Table. Number of clonally related isolate comparisons per sequence type (ST) with increasing time between isolates, in months. Within the pairwise comparisons all ST were the same except for an isolate with ST43, which was clonally related to an isolate of ST8567.**

| Months between isolates | | | | | | | | | | | | | |  |
| --- | --- | --- | --- | --- | --- | --- | --- | --- | --- | --- | --- | --- | --- | --- |
| ST | 1 | 2 | 3 | 4 | 5 | 6 | 7 | 8 | 9 | 10 | 11 | 12 | 13 | Total |
| 602 | 16 | 15 | 10 | 9 | 17 | 8 |  | 12 |  |  |  |  |  | 87 |
| 117 | 17 | 1 |  |  |  |  |  |  |  | 1 | 2 | 3 |  | 24 |
| 10 | 8 |  |  |  |  |  |  |  |  |  |  |  |  | 8 |
| 69 | 6 |  |  |  |  |  |  |  |  |  |  |  |  | 6 |
| 57 | 2 |  |  |  |  |  |  |  |  |  |  | 2 |  | 4 |
| 1158 | 1 |  |  |  |  |  | 2 |  |  |  |  |  |  | 3 |
| 58 | 1 | 2 |  |  |  |  |  |  |  |  |  |  |  | 3 |
| 88 | 3 |  |  |  |  |  |  |  |  |  |  |  |  | 3 |
| 1072 | 1 |  |  |  |  |  |  |  |  |  |  |  |  | 1 |
| 1304 |  |  |  |  |  |  |  |  |  |  |  |  | 1 | 1 |
| 1818 | 1 |  |  |  |  |  |  |  |  |  |  |  |  | 1 |
| 354 |  | 1 |  |  |  |  |  |  |  |  |  |  |  | 1 |
| 3778 |  |  |  |  |  | 1 |  |  |  |  |  |  |  | 1 |
| _43 - 8567_* |  |  |  |  |  |  |  | 1 |  |  |  |  |  | 1 |
| 4663 | 1 |  |  |  |  |  |  |  |  |  |  |  |  | 1 |
| 48 | 1 |  |  |  |  |  |  |  |  |  |  |  |  | 1 |
| 5183 | 1 |  |  |  |  |  |  |  |  |  |  |  |  | 1 |
| 752 |  |  |  |  |  |  |  |  |  |  | 1 |  |  | 1 |
| Total | 59 | 19 | 10 | 9 | 17 | 9 | 2 | 13 | 0 | 1 | 3 | 5 | 1 | 148 |
