# Supplementary material for: Decreasing prevalence of contamination with extended-spectrum beta-lactamase-producing Enterobacteriaceae (ESBL-E) in retail chicken meat in the Netherlands
Source: PLoS One. 2019 Dec 31;14(12):e0226828. doi: 10.1371/journal.pone.0226828 (PMC6938319; doi:10.1371/journal.pone.0226828)
Supplement: S6 Table — (DOCX) [file pone.0226828.s006.docx]

**S6 Table. Alternate multivariable models of frequency of clonality using wgMLST, excluding the time variable in alternative model 1 and using shorter time periods in the first year in alternative model 2.**

|  | Generalized linear model – Binomial – Robust error estimation | | | |
| --- | --- | --- | --- | --- |
|  | Alternate multivariable model 1 | | Alternate multivariable model 2 | |
|  | ARR | 95% CI | ARR | 95% CI |
| Method of farming |  |  |  |  |
| Between | ref |  | ref |  |
| Within | 1.41 | 0.97 - 2.05 | 1.34 | 0.92 - 1.95 |
| Supermarket chain |  |  |  |  |
| Between | ref |  |  |  |
| Within | 2.14 | 1.55 - 2.95 | 2.04 | 1.48 - 2.81 |
| Time between isolates |  |  |  |  |
| Month 1-4 |  |  | ref |  |
| Month 5-8 |  |  | 1.61 | 1.10 - 2.38 |
| Month 9-12 |  |  | 0.73 | 0.42 - 1.25 |
| Months >=12 |  |  | 0.08 | 0.04 - 0.16 |
| Abbreviations: ARR, adjusted relative risk; CI, confidence interval. | | | | |
