# Supplementary figures and images for: Decreasing prevalence of contamination with extended-spectrum beta-lactamase-producing Enterobacteriaceae (ESBL-E) in retail chicken meat in the Netherlands
Source: PLoS One. 2019 Dec 31;14(12):e0226828. doi: 10.1371/journal.pone.0226828 (PMC6938319; doi:10.1371/journal.pone.0226828)

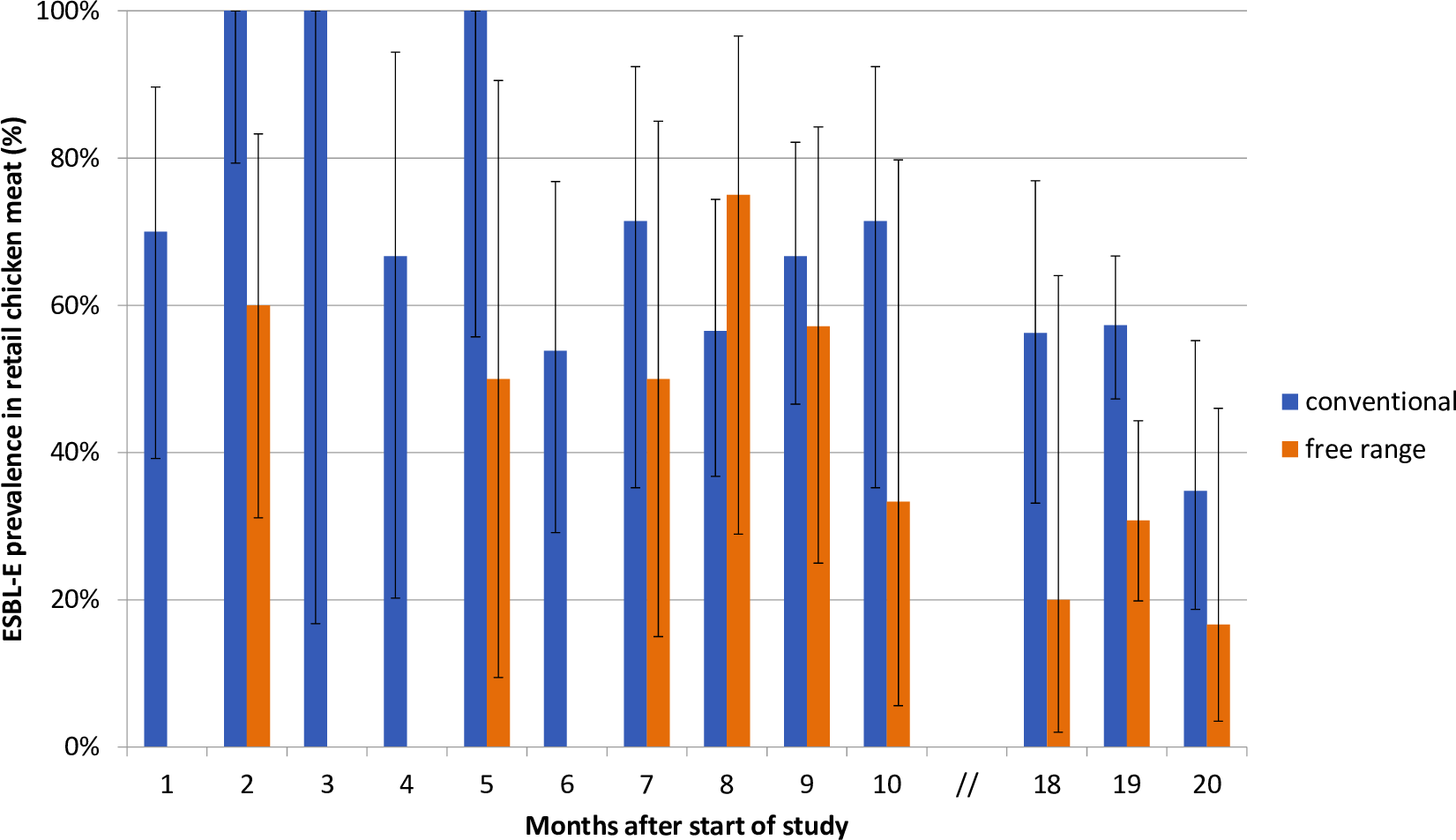

Supplement: S1 Fig — X-axis shows time in months after start of the study, error bars show 95% confidence intervals. (TIF) [file pone.0226828.s007.tif]

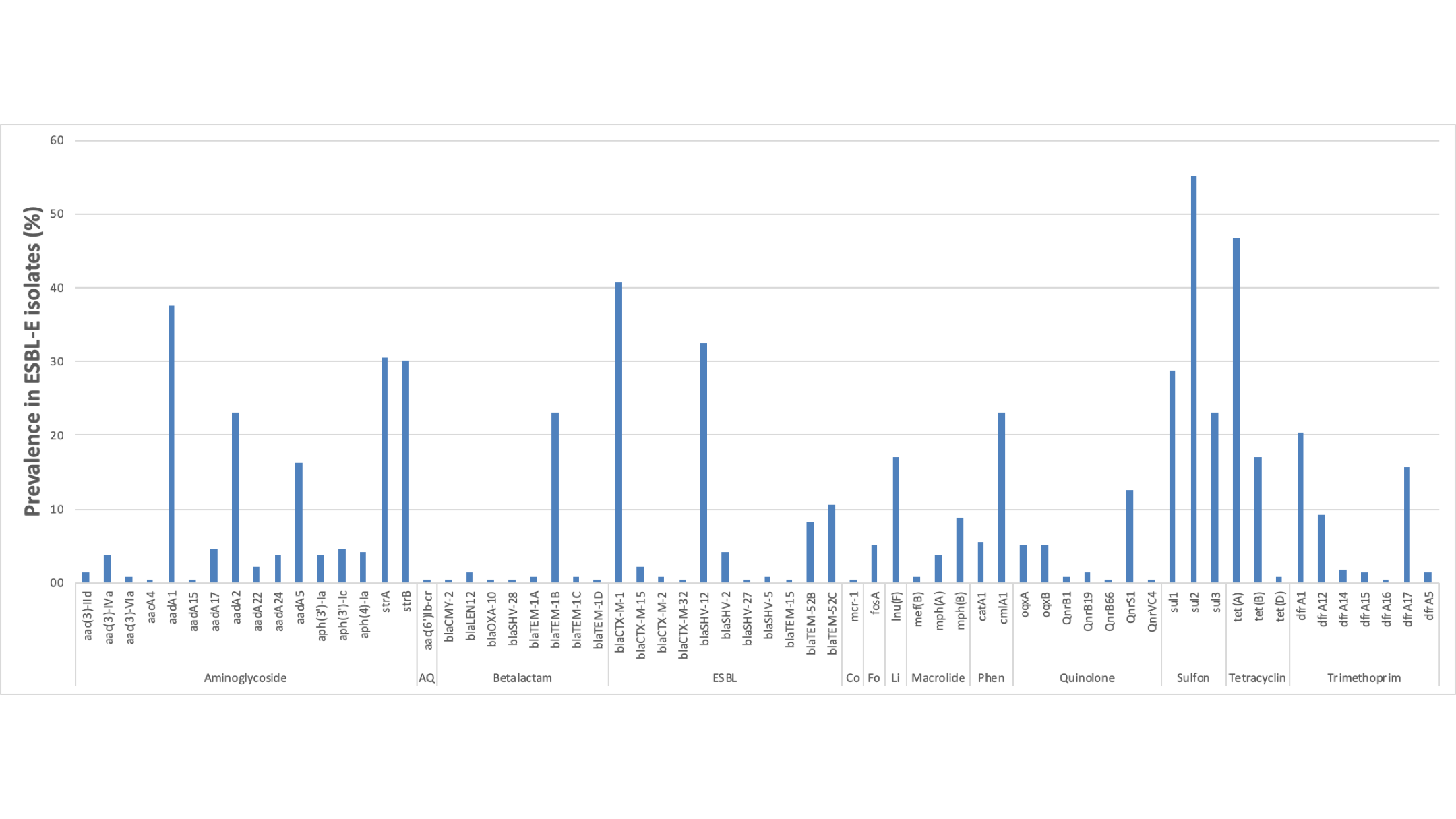

Supplement: S2 Fig — Abbreviations: Sulfon, sulfonamide; Phen, phenicol; Li, lincosamide; Fo, fosfomycin; ESBL, extended-spectrum beta-lactamase; AQ, aminoglycoside and quinolone. (TIF) [file pone.0226828.s008.tif]

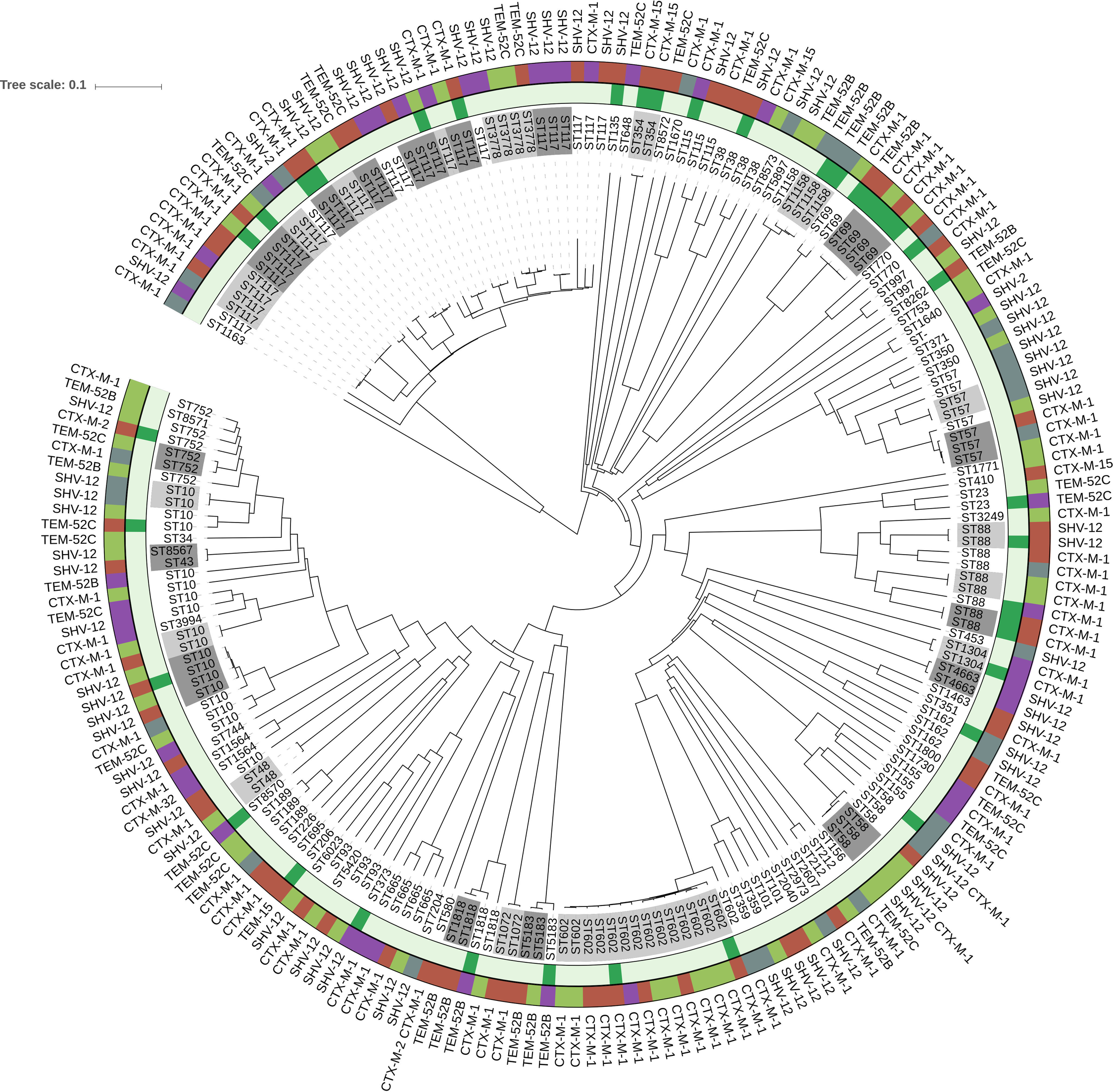

Supplement: S3 Fig — Legend, circles from inside out: conventional sequence type; shading in light or dark grey of the sequence type indicates clustering in whole genome multilocus sequence typing analyses; method of farming, light green is conventional and dark green is free range; supermarket chains: red SC4, light-green SC3, purple SC2, grey-cyan SC1; the outer most ring shows the detected ESBL genes in each isolate. (TIF) [file pone.0226828.s009.tif]

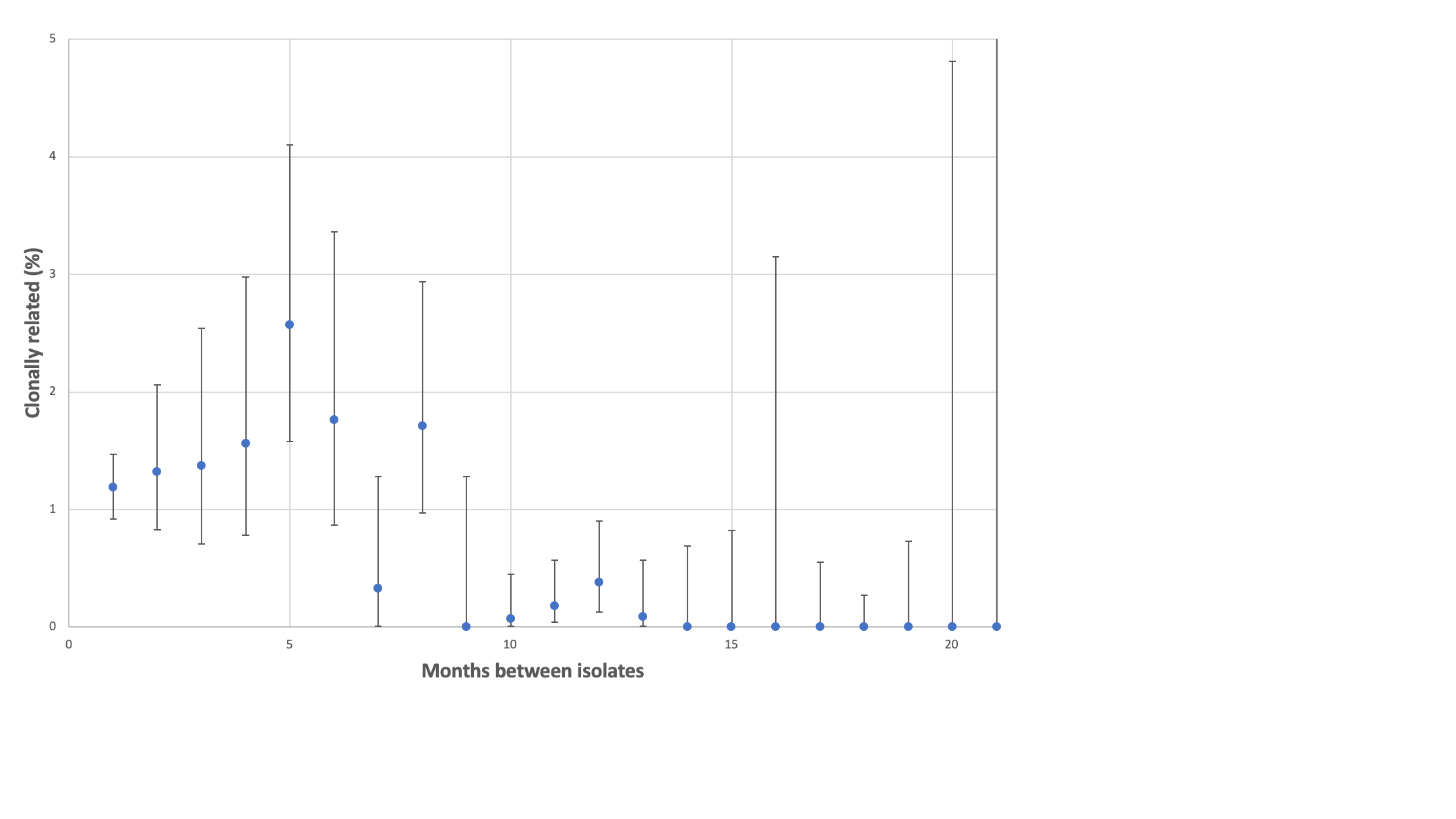

Supplement: S4 Fig — (TIF) [file pone.0226828.s010.tif]
