# Supplementary material for: Decreasing prevalence of contamination with extended-spectrum beta-lactamase-producing Enterobacteriaceae (ESBL-E) in retail chicken meat in the Netherlands
Source: PLoS One. 2019 Dec 31;14(12):e0226828. doi: 10.1371/journal.pone.0226828 (PMC6938319; doi:10.1371/journal.pone.0226828)
Supplement: Attachment — Submitted filename: Response to Reviewers.docx [file pone.0226828.s011.docx]

**Reference: PONE-D-19-22286
Title: Decreasing prevalence of contamination with extended-spectrum beta-lactamase-producing Enterobacteriaceae (ESBL-E) in retail chicken meat in the Netherlands**

Dear Mr. Johnson,

We kindly thank you and the reviewers for the critical appraisal of our manuscript and the constructive comments. We have tried to comply with the suggested changes and hope that with these changes you will consider the manuscript for publication in PLOS ONE.

Kind regards,

Pepijn Huizinga

**Journal Requirements:**

When submitting your revision, we need you to address these additional requirements.

1. Please ensure that your manuscript meets PLOS ONE's style requirements, including those for file naming. The PLOS ONE style templates can be found at

[http://www.journals.plos.org/plosone/s/file?id=wjVg/PLOSOne_formatting_sample_main_body.pdf](http://www.journals.plos.org/plosone/s/file?id=wjVg/PLOSOne_formatting_sample_main_body.pdf) and [http://www.journals.plos.org/plosone/s/file?id=ba62/PLOSOne_formatting_sample_title_authors_affiliations.pdf](http://www.journals.plos.org/plosone/s/file?id=ba62/PLOSOne_formatting_sample_title_authors_affiliations.pdf)

**Response:**

Different changes in the format have been applied to the manuscript. These include: using the structured level headings, indentation for paragraphs and adhering to the requested filenames.

2. In your Methods section, please provide additional details regarding how you chose the sample size for this study, e.g. power calculation.

**Response:**
**A previous study from the group published by Overdevest et al. had shown ESBL-E prevalence of up to 80% in chicken meat [14]. At the end of 2013 and beginning of 2014 a new set of samples was taken to see if it had remained high over time. Although it was high, it was lower than was previously found and we wanted to show a decreasing trend. The power was recalculated with knowledge of the phenotypic ESBL-E prevalence in the first period. To detect a decline of 15% with a power of 80% and alpha of 0.05 a total of 341 samples, with 199 samples in the second period had to be collected. Therefore, we consider this study sufficiently powered. The following sentence was added to the method section:**

*The sample size of the second period was calculated after the first collection period. To detect a decline of 15% in ESBL-E prevalence with a power of 80% and an alpha of 0.05 with 142 samples in the first period and an ESBL-E prevalence of around 68% in that first period, 199 samples had to be collected in the second sampling period.*

[Note: HTML markup is below. Please do not edit.]

Reviewers' comments:

Reviewer's Responses to Questions

**Comments to the Author**

1. Is the manuscript technically sound, and do the data support the conclusions?

The manuscript must describe a technically sound piece of scientific research with data that supports the conclusions. Experiments must have been conducted rigorously, with appropriate controls, replication, and sample sizes. The conclusions must be drawn appropriately based on the data presented.

Reviewer #1: Partly

Reviewer #2: Partly

2. Has the statistical analysis been performed appropriately and rigorously?

Reviewer #1: No

Reviewer #2: Yes

3. Have the authors made all data underlying the findings in their manuscript fully available?

The [PLOS Data policy](http://www.plosone.org/static/policies.action#sharing) requires authors to make all data underlying the findings described in their manuscript fully available without restriction, with rare exception (please refer to the Data Availability Statement in the manuscript PDF file). The data should be provided as part of the manuscript or its supporting information, or deposited to a public repository. For example, in addition to summary statistics, the data points behind means, medians and variance measures should be available. If there are restrictions on publicly sharing data—e.g. participant privacy or use of data from a third party—those must be specified.

Reviewer #1: No

Reviewer #2: Yes

4. Is the manuscript presented in an intelligible fashion and written in standard English?

PLOS ONE does not copyedit accepted manuscripts, so the language in submitted articles must be clear, correct, and unambiguous. Any typographical or grammatical errors should be corrected at revision, so please note any specific errors here.

Reviewer #1: Yes

Reviewer #2: Yes

5. Review Comments to the Author

Please use the space provided to explain your answers to the questions above. You may also include additional comments for the author, including concerns about dual publication, research ethics, or publication ethics. (Please upload your review as an attachment if it exceeds 20,000 characters)

Reviewer #1: The study of Huizinga et al. deals with the investigation of chicken meat concerning ESBL-producing Enterobacteriaceae in the Netherlands. Samples were conducted during two different periods and respective isolates were investigated using whole genome sequencing and wgMLST analyses. Furthermore, some statistics about possible phylogentic relationships were carried out. From their data the authers concluded a reduction in the prevalence of the resistant bacteria from 2014 to 2015 and found lower prevalence in meat from free-range chicken than in meat from conventional farms.
The laboratory methods used in the study are state of the art; however, there is a huge lack in information about the samples and the investigated "supermarket-chains" and, therefore, conclusions might be doubthly
1) the first sampling period started in December 2013 but the authors analysed 2014 to 2015. Did they included 2013 in the 2014 sample set?

**Response:**

**We fully agree with the reviewer and have added two sentences in the method section defining the periods from December 2013 to October 2014 as “period 2014” and the same for June 2015 until August 2015 as the “period 2015”. Naming the first period “period 2014” is not strictly correct, but in total 7 samples were purchased in 2013. Naming it “period 2013 – 2014” we believe will represent a less accurate picture. The sentence in the method section will be altered to the following:**

*A convenience sample of chicken meat samples were collected from December 2013 until October 2014 and will be referred to as “period 2014”, and from June 2015 until August 2015 this will be referred to as “period 2015”.*

2) it is discussed that two different periods were sampled (11 month vs 3 month). How many samples were taken each month? how many samples were from conventional (each month) and from free-range (each month)? this information is important to know if you really can conclude a reduction from sampling such different time periodes.

**Response:**

**We agree with the reviewer that the requested information is important. We have added a supplementary table, S1 Table, showing how many samples were taken per month, per supermarket chain and per method of farming. It is referred to in the result section:**

*A table showing the number of samples taken per month, per supermarket chain and per method of farming is shown in S1 table.*

3) How are the supermarket chains defined? Where did the chain started? At the farm? The slaughterhouse? The packaging? (A graphical overview would be helpful especially as it was stated that two chains were somehow combined) How many samples each chain were taken?

**Response:**

**We believe this question arises from a misunderstanding; with a ‘supermarket chain’ we mean a group of supermarkets such as Tesco in the UK or Kroger in the US. Samples were purchased from different supermarkets that belong to the same supermarket chain. The chain that the reviewer means is discussed in the discussion section of the paper and we called it ‘production chain’. We have asked multiple native speakers for a possible other term for supermarket chain but heard no option that was clearer. Also, Wikipedia refers to this as supermarket chain. If the editor or reviewer have an alternative suggestion we will be happy to use a different term. The amount of samples taken per supermarket chain is shown in table 1 and in the newly added S1 table.**

4) From Figure2 one would assume each chain comprises conventional and free-range products. It´s not likely that free-range and conventional were fattened at the same farm. Were exactly the same chains with comparable numbers of samples investigated during both periods?

**We believe that this question is based on the same misunderstanding as the previous question. Further data on how samples were purchased has been added in S1 table as requested in the reviewers second question.**

S1 Fig) This figure is highly missleading! Are the samples of 2013 included as well? And summarizing 3 month in two qaters (q6 and q7) indicate a much larger sampling periode than conducted. This should be changed to month and should also differentiate between free-range and conventional

**The study started in December 2013 and all samples are included in the figure. Due to low intensity sampling in the first ten months of the study it was decided to display the numbers per quarter. In our opinion, it shows the bigger picture better than the graph that shows percentages per month. To accommodate the reviewer, we have changed the figure in the manuscript.**

**With this figure we visually show the decrease in ESBL-E prevalence. Further stratification of the data in for instance free range and conventional farming decreases the clarity of the graph and the multivariable analyses gives this information.**

**We have added a quick draft of the requested figure for illustration purposes to this answer.**

S3 Fig) Legend/explanation of colors are missing

**Response:**

**The legend and explanation of the colours in the figure are present at the end of the manuscript as requested in the PLOS ONE authors guide. For S3 Fig it is the following:**

*S3 Fig. Neighbour-joining tree based on the wgMLST analysis of 204 ESBL-producing E. coli isolates cultured from retail chicken meat in the Netherlands, using a “pairwise ignore missing values” approach. Legend, circles from inside out: conventional sequence type; shading in light or dark grey of the sequence type indicates clustering in whole genome multilocus sequence typing analyses; method of farming, light green is conventional and dark green is free range; supermarket chains: red SC4, light-green SC3, purple SC2, grey-cyan SC1; the outer most ring shows the detected ESBL genes in each isolate.*

S4 Fig) what about the impact of the supermarket chain and the fattening conditions on the clonality? One would expect closer relations in the same chain. Therefore, samples should be analysed differently as indicated in table 4 and this figure can be excluded

**Response:**

**We agree with the reviewer that supermarket chain and fattening conditions have a relation on the frequency on clonality as is shown in table 4. However, the effect of time is profound and non-linear. To show this change over time we believe S4 Fig is very relevant and would rather not omit it. (We also use this figure in answers to reviewer 2.) We expect the reviewer would have preferred a stratified analysis, however, there is quite some clonality between the different supermarket chains (table 4 bottom half) and between the different fattening conditions. As such we believe the analysis has merit in its present form. If the reviewers do not agree with this analysis we prefer to remove it completely from the paper, but we would rather keep it.**

Reviewer #2: Overall, the manuscript is interesting, extremely relevant and discussion is very well-balanced with limitations included. Please consider the following suggestions to make this manuscript even stronger.


Major comments:

Methods section:
Sample collection- Needs more epidemiological information. Are these supermarkets representative of the whole Netherlands? How geographically dispersed are these supermarket chains?

**Response:**

**These supermarket chains are present throughout the Netherlands and have combined market share of 70%. We have added a sentence to the methods section.**

**“***The combined market share of the sampled supermarket chains in the Netherlands is around 70%.”*

Since these are chains, I assume there were multiple stores. How many stores per supermarket chain sampled?

**Response:**

**Unfortunately this information was not noted, but multiple stores per supermarket chain were visited. We do not expect any differences in ESBL-E prevalence in the different stores, as all of these supermarket chains are supplied from central warehouses. We cannot imagine significant differences between stores from the same supermarket chains. However, we did not investigate this.**

Two supermarket chains merged during study. So, were there 4 or 5 supermarkets to begin with?

**Response:**

**At the start of the study there was a supermarket chain *group* that consisted of two supermarket chains (Dutch market shares 17.4 and 1.1%). The smaller of these two chains was taken over and took the name of the larger supermarket chain. In total 22 samples from this smaller chain are in the study (total number of samples 352). We have added the underlined part of the following sentence to further clarify.**

“*Two supermarket chains, which were already part of the same group of supermarkets, merged during the study period and were analysed together as one supermarket chain as we assumed overlapping suppliers already before the official merger.”*

How was the sampling designed- random, convenient etc.?

**Response:**

**The sampling strategy was a convenience sample. This has been added to the first line of the method section.**

*“A convenience sample of chicken meat samples were collected from December 2013 until October 2014 and will be called period 2014, and from June 2015 until August 2015 this will be called period 2015.”*

Best before date was available but not used in any statistical analyses- would that have potentially confounded results e.g. samples closer to best before date might have higher levels of bacterial contamination.

**Response:**

**We agree that this could potentially be a confounder, but as all samples were before the ‘best before date’ they should be within norms for bacterial colonization. The current study did not look at bacterial loads and used a double broth enrichment technique (non-selective, followed by selective broth enrichment) so the original bacterial load would have had little impact except if the loads were extremely low.**

What do you mean by “biological chicken meat”?

**Response:**

**We thank the reviewer for this point, the term was translated incorrectly and should have been “organic” chicken meat. This was changed in the sentence:**

*Organic chicken meat was not sampled for this study.*

Microbiological methods: Did these EbSA plates contained cefotaxime or ceftazidime or were these plates split into 2 halves with one half containing cefotaxime and other half containing ceftazidime? If indeed, these plates carried just cefotaxime or just ceftazidime, then there is a bias in methods as using only one of these antimicrobials for ESBL screening can lead to differing results and false negatives.

**Response:**

**We thank the reviewer for pointing out something that could be interpreted incorrectly. For each culture one split plate was used with one half containing cefotaxime and the other half containing ceftazidime. We fully agree with the reviewer that using only one of the two cephalosporin’s will decrease the sensitivity of cultures considerably! We have altered the sentence in the materials and methods to the following sentence:**

*After a second overnight incubation, 10 μL of the TSB-VC was subcultured on an ESBL screening agar, EbSA (AlphaOmega, ‘s-Gravenhage, the Netherlands), consisting of a split McConkey agar plate containing cloxacillin (400 mg/L), vancomycin (64 mg/L) and on one half cefotaxime (1 mg/L) and the other half ceftazidime (1 mg/L).*

Whole genome sequencing and quality control: Please include the read lengths, chemistry versions and instrument type for MiSeq.

**Response:**

**We are not sure what the reviewer means with instrument type for the MiSeq as we believe MiSeq is the type. The other points were added to the following sentence:**

*Genomic DNA was prepared using the Nextera XT library preparation kit (Illumina, San Diego, United States). The libraries were sequenced on a MiSeq sequencer (Illumina, San Diego, United States) generating 250- to 300-bp paired-end reads using the MiSeq reagent kit v2 or v3 respectively*.

Statistical analyses: For analysis described in lines 187-194: There is a huge gap in time between October 2014 and June 2015 when sampling was not done. How was this missing time accounted for in the analyses? For example, if a clonal isolate was found in June 2014 and again in June 2015, then will the difference between these clones be considered as 12 months whereas there is a possibility that a similar clonal isolate was present but not sampled during this huge time-gap in sampling.

**Response:**

**We agree with the reviewer that a continuous sampling strategy would have been preferable and we should realise the effect of this shortcoming on this specific analysis. The analysis consists of pairwise isolate comparisons with a specific time between the two cultures. In the current analyses we focus on the percentage of the isolate comparisons that are clonally related. If in a certain time frame there are fewer comparisons it will decrease both clonal and non-clonal comparisons. We would expect the possible downside of not having samples and/or isolates from the missing periods is more in the precision of the analysis, due to less isolates, than we expect there to be any introduced bias due to periods with more or less clonality. Hence, we still stand by this analysis and believe there are enough cases in al time slots to show the presence of clonality. We add a figure showing the distribution of the time between isolates in this analysis. (Y-axis the frequency of pairwise comparisons, X-axis the time between isolates in days) We think it goes to far to include it in the paper or supplementary material but include it here to illustrate the point.**

Also, in lines 193-194: It is suggested that categories of time were chosen to coincide with frequency of clonality. I wonder if these models were built a-priori or on the basis of some statistical criteria. If models were built after looking at the data, then there might be a chance that models might be biased. Were there any sensitivity analyses performed e.g decreasing the periods from six months to 4 months or dropping some data and refitting the models to check for consistency of the results under various assumptions?

**Response:**

**We did not foresee the non-linearity of the time relationship. The categories were chosen with prior knowledge of the frequency of clonality, described in the method section. The reason for picking two half-year intervals and longer than 12 months can be seen in S4 Fig. Reasonably high clonality in the first 6 months, followed by lower rates of clonality in the second 6 months and after 12 months almost no clonality occurs. As the choice for this model was done made with knowledge of the outcome two alternative models were added. The following sentence was added to the method section: (answer continues in the questions posed by the reviewer in the result section)**

*As these choices were made with prior knowledge of the data, two alternative models were made excluding the time variable and using shorter time intervals in the first year.*

Results:
Please provide raw data (could be in supplementary form) about how many samples were collected per month, how many samples were collected per supermarket chain etc.

**Response:**

**We completely agree with the reviewer that this information was missing. We have added a table in the supplementary materials showing how many samples were collected per month per supermarket chain, per method of farming and referenced to this table in the manuscript:**

*The number of samples taken per month, per supermarket chain and per method of farming is shown in S1 Table.*

Table 1: How were the sample collected during December 2013 fit in this model? Are these included with 2014 isolates?

**Response:**

**We agree with the reviewer that this is not clear. In total 7 samples were purchased in 2013. We hope to have clarified this by describing the “period of 2014” as starting in December 2013 in the method section. We understand that this is not strictly correct but if the period will be named 2013 and 2014, we believe it will give a less correct picture of what was done.**

Table 2: there is no ST02 mentioned in the text. Is it ST602?

**Response:**

**It indeed is ST602 and has been corrected. We thank the reviewer for spotting this mistake!**

Table 3: Maintain consistency in gene nomenclature: bla should be italicized and CTX-M-1 should be a subscript.

**Response:**

**We refrained from subscripting the ESBL genes in the tables for readability but have now changed all the ESBL genes to subscript.**

Lines 286-287: Cite the actual median number of days and reference to the supplementary material.

**Response:**

**We have changed the sentence in accordance with the suggestion of the reviewer.**

*The median number of days between time of purchase of the samples that the clonally related isolates were cultured from was longer in ST602 (median 94 days, range 0 – 226 days), compared to ST117 (median 6.5 days, range 0 – 346 days) and ST10 (median 8 days, range 0 – 21 days), see S3 Table.*

Discussion: The biggest issue with both results and discussion section is the emphasis on decreasing prevalence of ESBL-E based on. A very short time period of 2 years. It has been noticed in several time-trend analysis that the prevalence of antimicrobial resistances vary (suddenly increase or decrease) considerably over short periods of time and sometimes without any apparent reasons; and longer time periods of 3-5 years are better to make any definitive claims. Hence, the authors are suggested to be include some verbiage to further highlight this limitation.

**Response:**

**We thank the reviewer for this point and the following sentences have been added to the limitation part of the discussion.**

*A final point of caution is the fact that the prevalence of ESBL-E has been known to vary over time and the timeframe in the current study is relatively short. However, the measured decrease in ESBL-E prevalence is considerable and the prevalence of ESBL/AmpC-producing Enterobacteriaceae has been shown to remain low. The MARAN reports show rates of contamination of retail chicken meat of 24% and 31.6% in 2016 and 2017 respectively which supports that our findings indicate a sustainable reduction of ESBL-E in retail chicken meat [refs in manuscript].*

Lines 317-319: Consider restructuring the sentence beginning with “ Since a number…”

**Response:**

**The sentence has been restructured to the following sentence:**

*These reports also describe the ESBL- and/or AmpC- (ESBL/AmpC) producing Enterobacteriaceae prevalence in retail chicken meat.*

Lines 319-323: Meaning of the sentence starting with “Although the selective…” not clear at all

**Response:**

**The original sentence was:** *Although the selective culture techniques used in MARAN and the reported unity (ESBL-E in current study and ESBL/AmpC-producing Enterobacteriaceae in MARAN) are different and hence, do not allow for direct comparison of the data with the results from this study, the decrease in ESBL-E prevalence in retail chicken meat is similar in both studies.*

**This has been changed to the following phrases:** *The reported results by MARAN are not directly comparable with the current study as the culture methods are different. Also, besides ESBL-E, AmpC-producing Enterobacteriaceae are included in the reported numbers. Despite these differences, the decrease in ESBL-E prevalence in retail chicken meat is similar in the MARAN reports compared to the current study.*

Lines 332-334: Did these “eight type of antibiotics” included cephalopsorins?

**Response:**

**One first generation cephalosporin was included, but the effect was found in other classes. However, this study used non-selective culture methods, which make it unsurprising that antibiotic classes which are used more frequently in poultry than cephalosporin’s show a larger effect. The sentence was changed to the following sentence:**

*In a study by Miranda et al. that looked at resistance rates to eight different types of antibiotics in randomly picked E. coli isolates, the resistance rates were higher in conventional chicken meat compared to organic chicken meat.*

Line 334- “With similar methods to previously mentioned study”. Which study is being referred to? Please cite.

**Response:**

**This sentence was clarified and the reference to the study mentioned in the sentence before it was removed:**

*Looking at resistance rates in randomly selected E. coli isolates to 12 types of antibiotics, under which three cephalosporin’s, Davis et al. found differences in resistance rates in turkey meat with different antibiotic use claims, but found that in chicken meat the brand of the meat had a larger effect than the antibiotic use claim*.

Lines 364-366: These sentences are critical as they imply other models were used using different categories for time. Please include these models in supplementary section.

**Response:**

**With this specific sentence we have tried to remove the emphasis from the point estimate instead of emphasizing it. When we added time as a continuous variable the results did not make sense. Therefore, the time variable was investigated further. It turned out that the frequency of clonality did not decrease in a linear fashion. Presenting this data would be incorrect, as the conditions for using a generalized linear model with time as a continuous determinant were not met.**

**An investigation was done for the effect of time, the results of which are shown in S4 Fig. For the first analyses, that is still currently in the main paper, we decided to categorize the time intervals in three segments with high, middle and low frequency of clonality.**

**The reviewer suggested an analysis using time periods of 4 months. We did this with 4 months periods for the first year and one category for longer than one year. A second alternative model was made in which the time variable was removed from the analysis. Both these models are added to a new supplementary table, S6 Table. In all these models the point estimates for method of farming and supermarket chain remain stable.**

**In our opinion, it is rather remarkable that frequency of clonality increases in the first months. We have added an extra table showing frequency of clonality per sequence type with increasing number of months between the isolates, S5 Table. Here one can see that all clonality in the months with the increase is caused by ST602. We are currently not sure how to interpret this and have added this to the result and discussion section.**

**The result and discussion section on this topic has been extensively edited. We include the full sections underneath this answer.**

Results:

*The general trend in frequency of clonality shows a decrease in clonality with increasing time interval between isolates as is shown in Table 4 and in more detail in S4 Fig****.*** *However, the first months show an increase in clonality with up to five months between the isolates showing the highest rates of clonality. All clonally related isolates with 3 – 5 months between the isolates belong to ST602, see S5 table for frequency of clonality per month per ST. No clonal relatedness was found in isolates more than 13 months apart. The frequency of clonality within supermarket chains was higher than the frequency of clonality between supermarket chains, see* *Table 4. This holds true with the exception of supermarket chain 3 and supermarket chain 4,* *for which the between supermarket chain frequency of clonality was higher than most other within supermarket chain comparisons. See Table 4 for all individual supermarket chain comparisons. No effect on the frequency of clonality within or between method of farming was observed. In the multivariable analyses within supermarket chain comparisons were twice as likely to be clonally related compared with between supermarket chain comparisons, adjusted RR of 2.0 with 95% CI 1.5 – 2.8. As time intervals were chosen with knowledge of the data, different models were tested, see S6 Table. Decreased time intervals of four months between the isolates in the first year showed higher clonality with 5 – 8 months between isolates compared to 1-4 months between the isolates. This was however followed by the expected decrease in clonality. Also, the time component was removed from the multivariable analyses. These changes to the model had little impact on the point estimates for supermarket chain or method of farming.*

Discussion:

*We were surprised by the relation of time between the isolates and the frequency of clonality. We expected a decrease over time, but found an increase in frequency of clonality up to five months between the isolates. After this increase in frequency of clonality it declines rapidly with the maximum time between clonally related isolates being 13 months. The increase in the frequency of clonality in the first months is almost solely caused by ST602. This highly clonal cluster stands out and the clonally related isolates have a longer median time between isolates compared to other clusters (ST10 and ST117). A possible explanation could be relatively low genetic variability in the sequence type. However, continued introduction to the food chain from a point source or temporary storage of a contaminated batch are other possibilities to explain the observation. Different options to cope with this time observation were tried in multivariable models that also looked at the effect of the supermarket chain and the method of farming on the frequency of clonality. In the different models the effect sizes of the latter two factors remained stable but the effect size of time between the isolates fluctuated with the different categorical options for time. We believe the key message on time between the isolates and clonality is that almost no clonality is seen in samples more than 12 months apart.*

*The second message from the clonality analysis is that isolates are twice as likely to be clonally related when the isolates are from within one supermarket chain, compared to isolates from different supermarket chains. This may be explained by overlapping production chains that give rise to more epidemiological relations between the isolates. Such relations could be isolates from chicken meat from the same farm, or possible contaminations from a common source in the processing of the meat. The higher frequency of clonality between supermarket chain 3 and supermarket chain 4 suggests a common source somewhere in the production chains. Clustering isolates closely matched in time could be due to batch contamination during processing of the meat, transmission between chickens or the chickens acquired the isolates from a common source. Clonal isolates cultured from samples collected months apart could have a wide range of possible sources of contamination. We could not verify hypotheses of where contamination or transfer may have occurred, as the production chain of the individual chicken meat samples was not accessible to us. However, combining this type of high-resolution typing data with precise knowledge of the flow of the products through the production chain and the possibility to go back and sample through that production chain could create the possibility to eliminate steps where contamination of meat products occurs.*

Conclusion:

*In pairwise isolate comparisons, clustering occurs more often within supermarket chains than between supermarket chains and clustering is absent in isolates cultured more than 13 months apart*.

Minor comments:
Line 40: Meaning of the sentence not clear due to use of “evaluable”

**Response:**

**The sentence was changed and a phrase added:**

*“In the current study, 352 chicken meat samples were investigated for the presence of ESBL-E using selective culture methods. Six samples were excluded due to missing isolates or problems obtaining a good quality sequence leaving 346 samples for further analyses.*”

Line 61: Should be “were” instead of “was”

**Response:**

**This has been changed in the text.**

Lines 64-66: For the sentence starting with “The epidemiology has…” – consider restructuring the structure. Not clear whether the epidemiology of pathogens have changed or related infections have changed

**Response:**

**This and the sentence before this line was changed to the following phrase, and a citation added:**

*“Originally ESBL-E infections were mainly a hospital-related problem with acquisition in hospitals or related to healthcare contact. This has changed in the past two decades with people that have had no healthcare contact also being rectal carriers of ESBL-E*” (Livermore 2007, Bunt 2019)

Lines 66-68: For sentence starting with “Research efforts…”- consider restructuring the sentence. Routes of transmission of what- pathogens? Genetic elements?

**Response:**

**The sentence has been changed to the following:**

*“Research efforts have focussed on uncovering routes of transmission and reservoirs of antimicrobial resistant microorganisms and resistance genes by using a one-health approach that includes humans, animals and the environment as an interconnected entity.”*

Lines 69-70: For sentence starting with “ESBL-E contamination…”- cite the year when these rates were estimated.

**Response:**

**This has been added.**

Line 100: should “and/or” instead of “and or”

**Response:**

**This has been changed**

Lines 416: should be “decreased” instead of “decreases”

**Response:**

**This has been changed and we thank the reviewer again for his constructive feedback. It has been much appreciated.**
